# Different Protein Sources Enhance 18FDG-PET/MR Uptake of Brown Adipocytes in Male Subjects

**DOI:** 10.3390/nu14163411

**Published:** 2022-08-19

**Authors:** Katarzyna Maliszewska, Edyta Adamska-Patruno, Katarzyna Miniewska, Witold Bauer, Angelika Buczyńska, Małgorzata Mojsak, Adam Kretowski

**Affiliations:** 1 Department of Endocrinology, Diabetology and Internal Medicine, Medical University of Bialystok, M. Sklodowskiej-Curie 24A, 15-276 Bialystok, Poland; 2 Clinical Research Centre, Medical University of Bialystok, M. Sklodowskiej-Curie 24A, 15-276 Bialystok, Poland; 3 Independent Laboratory of Molecular Imaging, Medical University of Bialystok, Żurawia 71A, 15-540 Bialystok, Poland

**Keywords:** brown adipose tissue, animal protein, plant protein, visceral adipose tissue, muscle mass, brain natriuretic peptide

## Abstract

Background: The unique ability of brown adipocytes to increase metabolic rate suggests that they could be targeted as an obesity treatment. Objective: The objective of the study was to search for new dietary factors that may enhance brown adipose tissue (BAT) activity. Methods: The study group comprised 28 healthy non-smoking males, aged 21–42 years old. All volunteers underwent a physical examination and a 75 g oral glucose tolerance test (75g-OGTT). Serum atrial and brain natriuretic peptide (ANP, BNP), PRD1-BF1-RIZ1 homologous domain containing 16 (PRDM16) and eukaryotic translation initiation factor 4E (eIF4E) measurements were taken, and 3-day food intake diaries were completed. Body composition measurements were assessed using dual-energy X-ray absorptiometry (DXA) scanning and bioimpedance methods. An fluorodeoxyglucose-18 (FDG-18) uptake in BAT was assessed by positron emission tomography/magnetic resonance (PET/MR) in all participants after 2 h cold exposure. The results were adjusted for age, daily energy intake, and DXA lean mass. Results: Subjects with detectable BAT (BAT^(+)^) were characterized by a higher percentage of energy obtained from dietary protein and fat and higher muscle mass (*p* = 0.01, *p* = 0.02 and *p* = 0.04, respectively). In the BAT^(+)^ group, animal protein intake was positively associated (*p*= 0.04), whereas the plant protein intake negatively correlated with BAT activity (*p* = 0.03). Additionally, the presence of BAT was inversely associated with BNP concentration in the 2 h of cold exposure (*p* = 0.002). Conclusion: The outcomes of our study suggest that different macronutrient consumption may be a new way to modulate BAT activity leading to weight reduction.

## 1. Introduction

The year 2009 saw a breakthrough on the presence of brown adipose tissue (BAT) in humans [1,2,3], even though the first reports were published in the 1980s [4,5]. Brown adipose tissue, due to unique protein uncoupling protein 1 (UCP1) in the inner mitochondrial membrane, has been acknowledged as a promising approach to increase energy expenditure [6]. The capacity of BAT to metabolize fat and raise metabolic rate may be used as a novel therapeutic mechanism to combat obesity and metabolic diseases [7].

Numerous studies have highlighted an association between BAT and body weight with an emphasis on the beneficial effect of brown adipocytes on decreasing central adiposity, which has a metabolically harmful status [8,9,10]. The activation of brown adipocytes plays a key role in thermogenesis. The most common BAT activators are cold exposure [11] and β-adrenergic agonist treatment [12]. Due to the side effects and inconvenience of such therapy, researchers have sought new possibilities to enhance BAT activity. One of the interesting ways to activate brown adipocyte seems to be the consumption of certain dairy macronutrients. Studies in animal models [13], as well as in humans [14,15], indicate that some specific food or macronutrient intake increases whole-body energy expenditure. Moreover, it has been noted that a high-fat diet induces the acquisition of brown adipocyte gene expression in white adipose tissue [16], whereas the consumption of carbohydrates increases the activity of the sympathetic nervous system (SNS) [17]. BAT consists of a dense network of sympathetic nerve endings. Noradrenalin is released after the activation of SNS; it binds to adrenoreceptors, mainly the B3 receptor, and the process of triglyceride (TAG) lipolysis in brown adipocytes is initiated [18]. The concept of diet-induced BAT thermogenesis is based on observations in rodents according to which increased SNS and BAT metabolic activities in diet-induced obesity are accompanied by lower weight gain than expected from the caloric intake, parallel with increased BAT activity and energy expenditure [19]. Moreover, recently published outcomes from animal studies have indicated the role of dietary protein in BAT activity with an emphasis on the influence of animal—especially dairy—protein in the expression of UCP 1 [20]. 

An interplay between protein intake and brown adipose tissue activity may explain this mutual relationship and support this role in weight maintenance. In light of the above, protein intake may directly or indirectly modulate BAT activity, and may be incorporated as a means to prevent obesity and reduce weight [21]. The majority of the US population meets or exceeds the minimum population recommendations for protein intake according to the Recommended Dietary Allowance (RDA 0.8 g/kg body mass) [22]. Dietary protein may come from different sources, and data from the National Health and Nutrition Examination Survey (NHANES) indicated the percentages of total protein intake derived from animal, dairy, and plant protein are 46%, 16%, and 30%, respectively [23]. This shows that the main source of protein consumption in the human diet is primarily animal protein (milk products and meat). The major element of protein found in bovine milk is casein, and it is the most commonly used milk protein in commerce today [24]. Milk proteins are a source of biologically active peptides, and they play a fundamental role in the body for functions relating to the uptake of nutrients and vitamins. Epidemiological studies have shown that diets based on dairy and vegetarian protein sources may protect against obesity [25]. 

Macronutrient consumption as a BAT activity modulator needs further investigation, especially regarding the possible underlaying molecular mechanism explaining this process. Recently, the role of a translation factor—eukaryotic translation initiation factor 4E (eIF4E)—was assessed in the obesity pathogenesis. The major cap-binding protein eIF4E was shown as a regulator of lipid homeostasis and diet-induced obesity [26]. Moreover, its role was also confirmed in glucose homeostasis and pancreatic Beta-cell function [27]. Outcomes from experimental studies have indicated that knockout mice (Eif4ebp1-/- manifest markedly smaller white fat pads than wild-type animals, and knockout males display an increase in metabolic rate [28]. These findings demonstrate that eIF4E could be a novel regulator of adipogenesis and metabolism in mammals; however, its role effect on brown adipocytes needs further investigation. Other stimuli for BAT include cardiac natriuretic peptides (NPs) and hormones that enhance lipolysis, particularly in response to cold, exercise, and food intake [29]. A-type natriuretic peptide (ANP), as well as B-type natriuretic peptide (BNP), apart from classical roles such as the regulation of the renal and cardiovascular systems, also control energy balance and glucose homeostasis, as well as thermogenesis [30]. ANP/BNP enhance triglyceride lipolysis as well as the uncoupling of mitochondrial respiration by inducing adipose tissue browning, which results in insulin resistance and the activation of the thermogenic program [31,32,33,34]. The regulation of BAT activity highlights the role of natriuretic peptides as a new regulator of food consumption and energy expenditure [35]. The transcriptional regulator involved in brown adipocyte regulation, PRDM16 (PRD1-BF1-RIZ1 homologous domain containing 16) needs further evaluation, because the data regarding its role in BAT activation are inconclusive. PRDM16 regulates a two-way transformation between skeletal myoblasts and brown fat cells. Moreover, its role inregulation of macronutrient intake and BAT activation seems intriguing [36]. 

The aim of the study is to evaluate the association between different dietary protein sources and brown adipose activity. We also assess the concentrations of natriuretic peptides (ANP, BNP), PRDM16, and eIF4E during cold exposure. 

## 2. Materials and Methods

### 2.1. Study Participants

Twenty-eight healthy, non-smoking Caucasian males aged 21–42 years (mean age 26.75 ± 5.11 years old) were enrolled in the study. Sixteen subjects had a normal body weight (BMI < 25 kg/m^2^) and twelve were obese/overweight (BMI ≥ 25 kg/m^2^). The presence of any comorbidities (such as hypo- or hyperthyroidis, cardiovascular disease, asthma, renal or liver failure) or the use of any medications (for example, beta blockers) that could have had an impact on the results led to participants being excluded from the research. We also excluded outdoor workers and shift workers from the study.

### 2.2. Study Procedures

#### 2.2.1. Screening of Subjects

During the screening visit, the medical histories of all subjects were obtained. For all participants, a physical examination, routine laboratory tests (TSH, hematology, creatinine, CRP, liver enzymes, K, Na), blood pressure measurements, an electrocardiogram (ECG), and an oral glucose tolerance test (OGTT) were performed. The OGTTs were performed with a 75 g glucose load according to World Health Organization (WHO) recommendations. 

#### 2.2.2. Dietary Assessments

All subjects completed a three-day food diary to evaluate macronutrient intake. Subjects compared their food portion sizes with the color photographs of each portion size and to weigh food, if possible. Daily total energy (kcal) and macronutrient (carbohydrate, fat and protein) intake were analyzed using Dieta 6 software (National Food and Nutrition Institute, Warsaw, Poland) [37,38]. Moreover, we divided dietary protein intake, depending on the source, into animal and plant proteins.

#### 2.2.3. Anthropometric Measurements 

A standardized method was applied to estimate the body height of the participants. Body weight was assessed in a standard way (InBody 720, Biospace, Seoul, Korea). Dual-energy X-ray absorptiometry (enCORE™, iDXA Lunar GE Healthcare, and InBody 720, Biospace Korea) was used to determine the body composition and body fat distribution. In further analyses, the following DXA measurements were estimated: visceral adipose tissue mass (VAT mass), visceral adipose tissue volume (VAT volume), the visceral adipose tissue percentage of body weight (VAT BW %), the visceral adipose tissue percentage of adipose tissue (VAT AT%), the android fat to gynoid fat ratio (DXA A/G ratio), free fat mass (FFM), and lean mass (Lean mass), IB AT-percentage (InBody adipose tissue percentage) content, IB SM mass (InBody skeletal muscle mass) content, IB SM mass percentage (InBody skeletal muscle mass) content, IB weight (InBody weight), IB VAT (InBody visceral adipose tissue). 

#### 2.2.4. Cold Exposure and PET/MR Scanning

On the second visit, in the fasting condition, all subjects underwent 2 h of cold exposure. Water-perfused blankets were used as part of the applied protocol for cooling. In the 60th and 120th min of cooling, the blood samples were collected. After this procedure, a fludeoxyglucose F 18 injection (18F-FDG) (4 MBq/kg of body mass) was administered intravenously and whole-body PET/MRI scanning (Biograph mMR 3T, Siemens Healthcare, Erlangen, Germany) was conducted during the autumn and winter periods.

Regions of interest (ROIs) were manually defined in fusion images composed of a summed dynamic FDG-18 PET image and magnetic resonance (MR). The software Carimas, developed at the Turku PET Centre in Finland, was applied for the image analyses. ROIs were drawn in image planes with a defined structure of brown adipose tissue and in the aortic arch in the time frame with the highest first-pass concentration of the tracer. Regional time–activity curves (TACs) were created, and glucose uptake rate data for the regions were estimated. The influx rate constant (Ki) of FGD-F18 for brown fat was assessed with the use of the Gjedde–Patlak model. A lumped constant (LC) value of 1.14 [39] was applied for all adipose tissues. The glucose uptake rate was calculated as follows: plasma glucose concentration × Ki × LC^−1^. The activation of BAT was defined as a glucose uptake rate higher than 2.0 µmol × (100 g^−1^) × min^−1^, which was chosen after a visual interpretation of PET images and the determination of the BAT glucose uptake rate at warm conditions, where it was always lower than 1.7 µmol × (100 g^−1^) × min^−1^ [40]. Participants in whom BAT was detected were enrolled in the BAT-positive group (BAT^(+)^), while subjects without detectable BAT in PET/MR images were matched to the BAT-negative group (BAT^(−)^). 

#### 2.2.5. Resting Metabolic Rate Measurements

On the second visit, during the colling procedure, a whole-body resting energy expenditure (REE) was estimated by a computed open-circuit indirect calorimetry method based on oxygen consumption and carbon dioxide production. The 30 min long measurements of resting O_2_ uptake and resting CO_2_ production were performed at the baseline (−30 min to 0 min) and every 30 min until 120 min of cold exposure, using a ventilated canopy system Vmax Encore 29n (Viasys HealthCare, Yorba Linda, CA, USA). 

#### 2.2.6. Blood Collection and Biochemical Measurements 

During the cooling, blood samples were taken and stored in −80 °C for further analyses. Serum ANP and BNP concentrations were evaluated using an enzyme-linked immunosorbent assay (ELISA) (ELISA Kit For Atrial Natriuretic Peptide (ANP), ELISA Kit For Brain Natriuretic Peptide (BNP); Cloud-Clone Corp., Wuhan, Hubei 430056, China, CEA225Hu, CEA541Hu, respectively) according to the manufacturer’s protocols.

For the purpose of the eIF4E and PRDM16 measurements, the cell lysates delivered from the red blood cells from whole blood were performed in line with the manufacturer’s instructions (Roche, 68305 Mnnheim, Germany, 11814389001). The eIF4E and PRDM16 cell lysate concentrations were measured using an ELISA method (Human eIF4E SimpleStep ELISA Kit, Abcam, Inc, Toronto, ON, Canada, ab214564; Enzyme-linked Immunosorbent Assay Kit for PR Domain Containing Protein 16 (PRDM16); Cloud-Clone Corp., Wuhan, Hubei 430056, China, SEP333Hu). Samples and controls were randomized, and then measured in the same run using a blind analysis method. 

ANP, BNP, eIF4E, PRDM16 were measured at baseline (fasting) and after 60 min and 120 min of cold exposure.

The colorimetric methods of the Cobas c111 analyzer (Roche Diagnostics, Basel, Switzerland) were used to measure the serum glucose concentrations. Samples and controls were measured in the same run using the blind analysis method.

### 2.3. Statistical Analyses

Numerical data have been summarized with a number of observations (N), arithmetic means, and standard deviations (SD). For categorical data, we present the number of observations and frequencies. We divided the study participants into two groups depending on the presence of BAT: BAT^(+)^ and BAT^(−)^. Continuous parameters were examined for normality using the Shapiro–Wilk test and through visual inspection. We studied the homogeneity of variance across the studied groups using the Levene test. We used non-parametric tests for response variables that failed these two tests. The differences between BAT groups and selected responses were compared using the analysis of variance (ANOVA) or Kruskal–Wallis tests for numerical variables, with Tukey or Dunn post hoc tests with a Holm *p*-value adjustment, respectively (in cases where multiple pairwise tests were performed or when there were multiple grouping variables). To study the hypothesis that there is a significant association between the presence of BAT and body composition and the hypothesis that daily macronutrient intake significantly alters BAT activation, we studied its association using multivariate linear regression models. These models were adjusted for age, daily kcal intake, and DXA lean mass. 

Huber–White robust standard errors (HC1) were calculated. The fit of the models was estimated using R-squared values and adjusted R-squared values. Some of the models were optimized using a backward stepwise elimination based on the Akaike Information Criterion (AIC). The statistical significance level was set at 0.05 for all two-sided tests and multivariate comparisons. All calculations were prepared in R (R version 4.0.2) [41].

### 2.4. Ethics

The study protocol was authorized by the local Ethics Committee of Medical University of Bialystok, Poland (R-I-002/233/2015). Written informed consent was gathered from all participants before inclusion to the study. The procedures were performed accordingly to the Helsinki Declaration of 1975, as revised in 1983. 

## 3. Results

**Figure 1:**
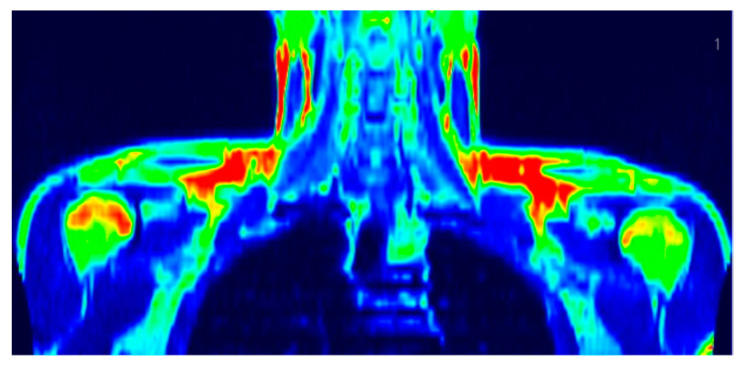
A PET/MR scan image of subject with brown fat depots in supraclavicular regions.

The study group characteristics have been published previously [42]; briefly, the BAT^(+)^ group (Figure 1) consisted of 18 subjects who were younger (mean age 24.7 years old vs. 30.3 years old *p* = 0.006) and slimmer (BMI 25 kg/m^2^ vs. 28 kg/m^2^ *p* = 0.1) compared to the BAT^(−)^ group. 

**Figure 2:**
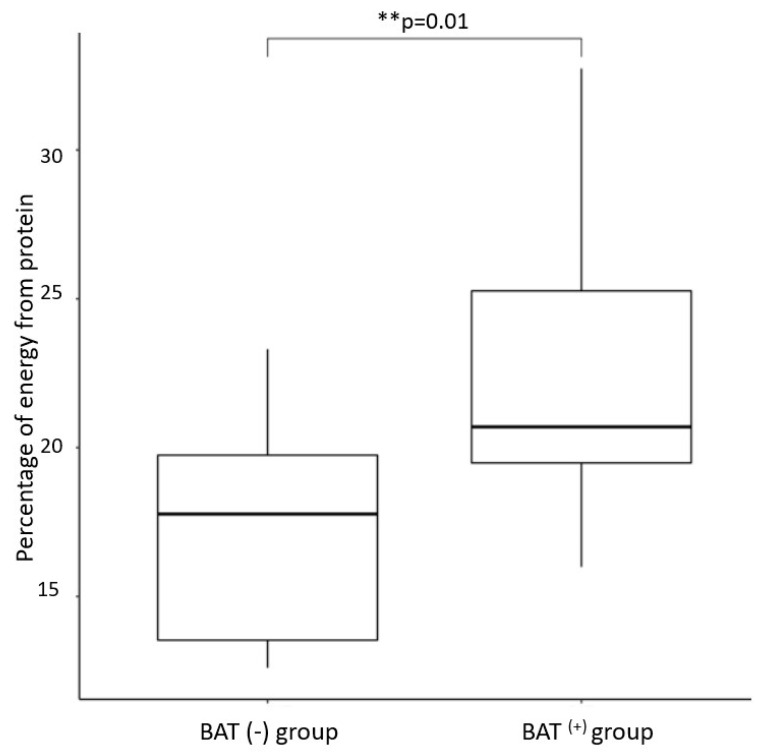
Percentage of daily energy intake from protein in subjects depending on BAT status ** *p* = 0.01.

**Figure 3:**
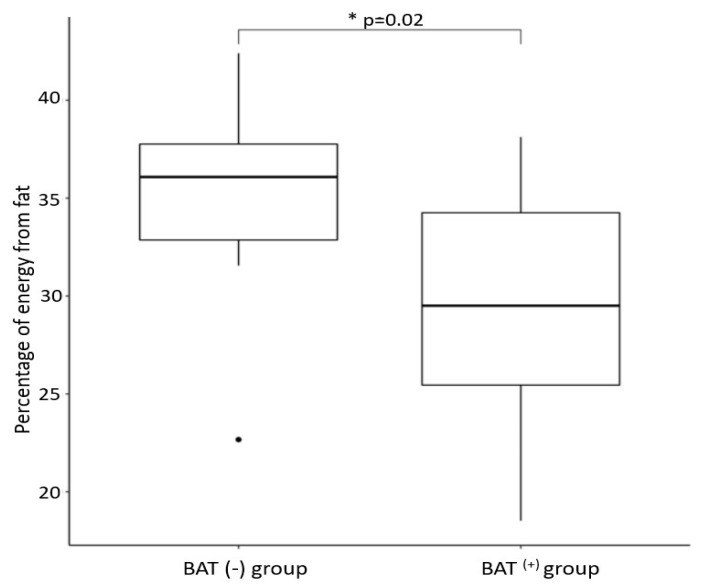
Percentage daily energy from fat in subjects depending on BAT status * *p* = 0.02.

The BAT(+) group was characterized by a higher percentage of daily energy intake obtained from protein and a lower percentage of daily energy obtained dietary fat (*p* = 0.01, *p* = 0.02, respectively, after age, daily energy intake, and DXA lean mass) (Figure 2 and Figure 3). We did not notice any significant differences in percentage energy obtained from carbohydrate consumption between the studied groups. Additionally, the groups did not differ in general daily macronutrient intake assessed in grams/day, as mentioned previously [42]. 

Moreover, subjects with detectable brown adipose tissue had a higher percentage of muscle mass (*p* = 0.04, after age, daily energy intake, and DXA lean mass adjustment) assessed by the bioimpedance method. Additionally, the group BAT ^(+)^ differed statistically significantly from BAT^(−)^ in the percentage content of visceral fat (*p* = 0.05 age, daily energy intake, and DXA lean mass adjustment) (Table 1). 

The outcomes based on multiple linear regression models showed in the BAT^(+)^ group that animal protein intake was positively associated with BAT activity (*p* = 0.04, Est. 0.01, R^2^ = 0.2467, after adjustment for age, daily energy intake, and DXA lean mass), whereas in the same group, the plant protein intake negatively correlated with BAT activity (*p*= 0.03, Est. −0.05, R^2^ = 0.33, after adjustment for age, daily energy intake, and DXA lean mass). Moreover, we noticed a positive association between a percentage energy obtained from dietary protein and BAT volume (Est. 3390.26941, R^2^ = 0.27, *p* = 0.02) and a negative association between the percentage of energy obtained from dietary carbohydrate and BAT volume (Est. −3394.07359, R^2^ = 0.39, *p* = 0.03 after age, daily energy intake and DXA lean mass). 

We sought for any other discrepancies between BAT^(+)^ and BAT^(−)^ groups evaluating the concentrations of following factors: ANP, BNP PRDM16 and EIHF4 at baseline (fasting) and during 2 h of cold exposure, but we did not observe any statistically significant differences between the studied groups. When multiple linear regression models were implemented to test independent determinants of the BAT ^(+)^ group, we noticed that the presence of BAT was negatively associated with BNP concentration in 2 h of cold exposure and delta BNP (Est. −24.26925, R^2^ = 0.39, *p* = 0.002 and Est. −26.33635, R^2^ = 0.37, *p* = 0.01, respectively after age, daily energy intake, and DXA lean mass). 

## 4. Discussion

In our study, we investigated the associations between dietary factors and brown adipose tissue activity and volume. These results indicate that the intake of different types of protein may influence BAT activity. This work’s outcomes show that subjects with detectable BAT are characterized by a higher percentage of daily energy obtained from dietary protein and fat. Moreover, we noticed a positive association between BAT activity and animal protein intake, as well as a negative correlation with plant protein intake. Additionally, in subjects with detectable brown adipose tissue, a skeletal muscle percentage content positively correlated with increased 18-FDG uptake by brown adipocytes, and the BAT^(+)^ group, as we reported previously [42], was characterized by a lower amount of total adipose fat content and visceral fat deposition. Additionally, in individuals with detectable brown adipose tissue, a concentration of BNP in 2 h of cold exposure was negatively associated with 18 FDG uptake in BAT. Our previously analyses in the same study group showed no statistically significant differences in daily kcal intake or REE [42].

High protein diets are widely known as an efficient tool to attenuate obesity [43]. High protein intake (25–35% of total energy), through a greater satiating effect, causes a reduction in energy consumption [44] leading to weight reduction [45] and changes in body composition [46]. In the meta-analysis of 24 trials, it was found that a high-protein diet ( 1.07–1.60 g·kg^−1^·d^−1^), compared with an energy-restricted standard protein diet, resulted in beneficial reductions in body weight, fat mass and triglycerides, and in a mitigated decrease in free fat mass and resting energy expenditure as well [47]. Additionally, greater satiety was reported in most of the analyzed trails [47]. A systematic review and meta-analyses of 74 randomized controlled trials (RCTs) compared higher- versus lower-protein diets using a 5% difference in total energy intake, and showed that high-protein diets caused a greater weight loss after 3 months compared to low-protein diets [48].

One of the explanations of sustained satiety after protein consumption is their impact on the release of peptide hormones from the gastrointestinal tract, such as glucagon-like peptide 1 (GLP-1), peptide YY (PYY), and cholecystokinin (CCK) [49]. These hormones and vagal afferent fibers regulate food consumption by acting on regions in central nervous system responsible for energy homeostasis: the brainstem, the hypothalamus, and brain regions related to reward and motivation [50]. Apart from sustained satiety in the process of weight reduction related to protein consumption, additionally, an increased metabolic rate is observed compared to carbohydrate or fat intake [51]. Macronutrient consumption increases energy expenditure due to nutrient processing such as digestion, absorption, transport, metabolism, and the storage of nutrients. Moreover, 24 h measurements of energy expenditure indicated increased metabolic rates in participants consuming the high-protein diet compared to standard protein intake (36% energy from protein vs. 15% energy from protein) [52]. Additionally, a recently published intervention study evaluating the effects of fat or protein overfeeding on energy expenditure in 25 participants indicated that excess energy from protein, but not fat, acutely stimulated the energy expenditure and sleep energy expenditure of 24 of the participants [53]. 

Brown fat, due to unique UCP1 in the inner membrane of mitochondrium, increases energy expenditure. The activation of brown adipocytes dissipates energy, leading to heat production and the promotion of a negative energy balance [6]. The active BAT inversely correlates with BMI, central obesity and visceral adipose tissue [3,54]. Outcomes from animal studies have indicated that the dietary protein can affect the gene expression of UCP1 in brown adipocytes [55,56]. It was noted that an increased intake of dietary protein was associated with an increased expression of UCP1 and other markers of a brown-like phenotype in white adipose tissue [57,58]. In some studies, despite an undetectable induction of UCP1 mRNA expression, the increased levels of UCP1 protein using immunohistochemistry and Western blotting in high-protein-fed mice was observed [20].

Studies in animals have shown that anti-obesogenic properties may differ dependently of protein sources. In mice fed with casein, soy, cod, beef, chicken or pork as protein sources, different impacts on obesity development were observed [20,59]. Mice fed with pork protein were more obese than casein-fed mice and relative to casein, the pork-based feed induced a substantial accumulation of fat in classic interscapular brown adipose tissue accompanied by decreased UCP1 expression. Casein was the most effective from all proteins at preventing weight gain and the accretion of adipose mass. It seems to have unique role in preserving classical brown morphology in interscapular brown adipose tissue and high UCP1 expression, even at thermoneutrality [20]. The role of casein in BAT activation was also supported by Chinese authors Hong Zhong et al., who showed that ß-casein-derived peptides (TPDHM1) in milk promote brown adipocyte energy metabolism. The thermogenic effect of TPDHM1 was possibly mediated by the p38-mitogen-activated protein kinase (p38-MAPK) signaling pathway. The inhibition of the p38-MAPK by adezmapimod (SB203580) abolished the UCP-1 expression induced by TPDHM1. Summarizing, the casein peptides induced UCP-1 expression (unpublished data; ADA 2020 poster presentation, Diabetes 2020 June; 69 (Supplement 1): (https://ada.scientificposters.com/epsAbstractADA.cfm?id=1), accessed on 13 June 2020).

In our study, we could only divide proteins into plant and animal sources, and we were not able to divide animal protein into more detailed sources, nor evaluate the families of proteins of diet, due to the relatively small sample size, and thus this needs further investigation. Nevertheless, the above-mentioned papers, although performed on animal models, support our results that different sources of protein intake may enhance BAT activity in different ways. 

Additionally, other data from the literature indicate the additional mechanism of brown adipose tissue activity through protein consumption. The regulation of energy expenditure and BAT thermogenesis can be also regulated by the vagal afferent neuron glucagon-like peptide-1 receptor (VAN GLP-1R). As mentioned above, a protein intake increases satiety through gut homes such as GLP-1, this gut–brain–BAT connection supports mutual dependence [60]. However, a number of studies suggest that brown adipose tissue activity is also involved in control energy balance due to GLP-1 receptor signaling [61,62,63]. 

In our study, we hypothesized that other molecules may be involved in energy balance regulation. We noticed that the concentration of BNP during 2 h of cold exposure was inversely associated with BAT activity. Atrial natriuretic peptides (ANPs) and brain natriuretic peptides (BNPs) have been already found to be involved in the regulation of BAT thermogenesis and the browning of white adipose tissue (WAT) [64]. Cold temperature exposure increases ANP/BNP in serum and the expression of natriuretic peptide receptors (NPRs) in BAT [65]. These findings are in in agreement with our results. Intriguing results came from another study concerning C-type natriuretic peptide-null mice, in which UCP1 mRNA expression in the brown adipose tissue was two-fold increased, oxygen consumption was significantly increased, and food intake of upon ad libitum feeding and refeeding after 48 h starvation was reduced by 21% and 61%, respectively, as compared with C-type natriuretic peptide (CNP)-mice [35]. This study implies that CNP decreases energy expenditure in brown fat by altering sympathetic nervous system activity, possibly under the control of the hypothalamus.

Regarding the unexpected function of eIF4E noticed in high-fat-diet obese animals, we evaluated a possible effect of major cap-binding protein eIF4E in our BAT(+) individuals. Conn CS et al. showed that, after the lipid overload, select networks of proteins involved in fat deposition were modified in eIF4E-deficient mice. The inhibition of eIF4E phosphorylation genetically and by a potent clinical compound restrained weight gain following the intake of a high-fat diet [26]. We assessed the concentration of eIF4E in our patients during 2 h of cold exposure, but we did not notice any differences between the groups at baseline and during the cold exposure. Additionally, we did not see any significant differences in PRDM16 concentration among subjects in terms of BAT activity. The major role of PRDM16 in adipose tissue is to induce a thermogenic program in subcutaneous WAT and induce browning in white fat [66]. In 2019, Wu L. et al. noticed that the transcription of PRDM16 is dependent on other factors, such as berberine (BBR) administration, which is a diarrhea drug [67]; therefore, we hypothesized that the macronutrient intake may have an impact as well, and more studies are needed to investigate this possible association. 

Dietary proteins play a fundamental role in the various anabolic processes of the body [68]. All dietary animal protein sources are seen to be complete proteins because they contain all of the essential amino acids. A profound increase in protein synthesis was observed after the high-animal-protein diet compared to the high-plant-protein diet [69], which resulted in greater gains in lean body mass [70]. The muscles utilize more than two-thirds of excess glucose disposal after intaking a meal; moreover, they are responsible for nearly all non-oxidative glucose storage as glycogen under hyperinsulinemia conditions [71]. A decline in muscle mass contributes to insulin resistance, finally leading to type 2 diabetes development [72]. In our study, we noticed that subjects with detectable brown adipocytes were characterized by a higher percentage of muscle mass content. Protein consumption, especially with animals as the source of protein intake, followed by increased 17FDG uptake by brown adipocytes, was associated with favorable metabolic profile. Those subjects were characterized by lower visceral fat accumulation and higher muscle mass. These outcomes suggest the beneficial effect of animal protein intake on brown adipocyte activity. Long-term follow-up observations are needed because observed associations may be beneficial in terms of the prevention of metabolic complications such as obesity and type 2 diabetes.

As far as we know, only a few other papers concern the association between macronutrient intake and BAT activity. Recently published results, based on 102 young adults in whom energy intake was estimated via an objectively measured ad libitum meal and habitual dietary intake, suggest that BAT does not play any important role in the regulation of energy consumption in either women or men [73]. In 2020, other studies were performed to test whether BAT thermogenesis is activated after single meal intake [74]. Saito at al. found a meal-induced metabolic activation of BAT in rats; that is, 30 min after the oral intake of a liquid meal, glucose utilization and fatty acid synthesis were increased in intact BAT, but to a much lower extent in surgically denervated BAT [75]. In 2017, the critical role of UCP1 in diet-induced thermogenesis (DIT) was proven by a simultaneous 24 h recording of food intake and oxygen consumption in UCP1-deficient mice [76]. Vrieze et al., in 2012, unexpectedly found a reduction in FDG uptake in the postprandial state into BAT compared with that after overnight fasting [77]. Vosselman et al. also reported, in 2013, that postprandial FDG uptake into BAT was much lower than cold-induced uptake, whereas whole-body EE was comparable [78]. Heenan et al. in a recently published meta-analysis concluded that diet is unlikely to affect BAT activity [79]. Additionally, the choice of tracer is very important in terms of BAT thermogenesis. FDG uptake into BAT could be used as a key factor of BAT activity under certain restricted conditions, while measuring oxygen uptake using ^15^O[O_2_]-PET and blood flow using ^15^O[H_2_O]-PET are more definitive indicators of thermogenesis and mitochondrial substrate oxidation. A number of studies have indicated an increase in oxygen consumption and blood flow, indicating that brown fat makes a contribution to diet-induced thermogenesis, at least in part, in humans [80,81,82].

We conducted a preliminary study, and our results suggest that this subject may be worth further investigation. Although our outcomes are intriguing and cutting-edge, our study has some important limitations. The study population comprised young, healthy male adults; hence, it remains unknown whether these findings would match with older and female populations. Additionally, the small sample size of the studied group could be one of the barriers, due to some financial limitations of our study. PET/MR studies generate very high costs; therefore, we could not include a higher number of subjects. For our study, we needed to purchase the tracer from a different center, which also increased the cost of the study and limited the number of volunteers. The results from our study suggest that BAT activity may be associated with sources of diet protein, which could be useful in obesity prevention and treatment; however, considering our study limitations, it should be investigated on larger populations. Additionally, intervention studies with specific nutrient consumption should be carried out, in addition to research investigating molecular factors and pathways potentially regulating brown fat activity.

It should also be highlighted that although 18F-FDG is currently the most available tracer for assessing human brown fat volume, it has serious limitations as a tool for assessing BAT metabolic activity. Another important fact is that brown adipocytes are mingled with white adipose tissue. Therefore, through PET detection, BAT regions could consist of both brown and some white adipocytes. Moreover, data from the literature [83] indicate that BAT prefers fatty acids over glucose as a metabolic fuel in the first stage of thermogenesis. Taking this into consideration, it is essential to evaluate BAT activity with a radioactive tracer combined with a different tracer. It is worth noting that cooling might be suboptimal for some subjects, especially for obese subjects, thus resulting in false negative results related to BAT activity. In our study, water-perfused blankets were used, and many scientific studies with cold exposure to large skin areas, such as via water-perfused suits or vests, seem to show minor fluctuations in BAT activation [84].

## 5. Conclusions

In our study, an enhanced 18-FDG uptake was observed in volunteers characterized by a higher percentage of daily energy intake obtained from animal protein intake. The outcomes of the study suggest that different protein sources may influence BAT activity, and more favorable body composition profiles as well.

## Figures and Tables

**Table 1:** Characteristics of BAT^(+)^ and BAT^(−)^ male group.

Variables ¹	BAT^(+) 2^	BAT^(−) 3^	*p*-Value ^4^
Age (years)	24.7 ± 2.4	30.3 ± 6.7	0.006
BMI < 25 kg/m^2^ (N)	12	4	
BMI > 25 kg/m^2^(N)	6	6	
IB weight	86.7 ± 16.5	94.01 ± 15.0	0.1
IB AT percentage (%)	18.4 ± 6.5	22.5 ± 5.9	0.06
IB SM mass	39.9 ± 5.5	41.1 ± 5.2	0.1
IB SM percentage	46.4 ± 3.7	44.0 ± 3.3	0.04
IB VAT (cm^2^)	71.6 ± 41.3	99.3 ± 38.6	0.05
Percentage of daily energy obtained from protein	22.6 ± 4.9	17.2 ± 3.9	0.01
Percentage of daily energy obtained from fat	29.3 ± 5.2	34.9 ± 6.1	0.02
Percentage of daily energy obtained from carbohydrates	46.0 ± 6.7	45.4 ± 7.6	0.8

^1^ Represented by means ± SD. ^2^ BAT^(+)^—a group of subjects in whom brown adipose tissue was detected; ^3^ BAT ^(−)^—a group of subjects without detectable brown adipose tissue; ^4^ *p*-value—adjusted for age, daily energy intake, and DXA lean mass; IB AT percentage (InBody adipose tissue percentage) content, IB SM mass (InBody skeletal muscle mass) content, IB SM mass percentage (InBody skeletal muscle mass percentage) content, IB weight (InBody weight), IB VAT (InBody visceral adipose tissue).
